# Reduced metabolic capacity in fast and slow skeletal muscle via oxidative stress and the energy‐sensing of AMPK/SIRT1 in malnutrition

**DOI:** 10.14814/phy2.14763

**Published:** 2021-03-02

**Authors:** Takumi Hirabayashi, Ryosuke Nakanishi, Minoru Tanaka, Badur un Nisa, Noriaki Maeshige, Hiroyo Kondo, Hidemi Fujino

**Affiliations:** ^1^ Department of Rehabilitation Science Kobe University Graduate School of Health Sciences Kobe Japan; ^2^ Department of Rehabilitation Nose Hospital Kobe Japan; ^3^ Faculty of Rehabilitation Kobe International University Kobe Japan; ^4^ Department of Rehabilitation Science Osaka Health Science University Osaka Japan; ^5^ Department of Food Science and Nutrition Nagoya Women’s University Nagoya Japan

**Keywords:** malnutrition, metabolic capacity, oxidative stress, SIRT1

## Abstract

The effects of malnutrition on skeletal muscle result in not only the loss of muscle mass but also fatigue intolerance. It remains unknown whether the metabolic capacity is related to the fiber type composition of skeletal muscle under malnourished condition although malnutrition resulted in preferential atrophy in fast muscle. The purpose of the present study was to investigate the effects of metabolic capacity in fast and slow muscles via the energy‐sensing of AMPK and SIRT1 in malnutrition. Wistar rats were randomly divided into control and malnutrition groups. The rats in the malnutrition group were provided with a low‐protein diet, and daily food intake was limited to 50% for 12 weeks. Malnutrition with hypoalbuminemia decreased the body weight and induced the loss of plantaris muscle mass, but there was little change in the soleus muscle. An increase in the superoxide level in the plasma and a decrease in SOD‐2 protein expression in both muscles were observed in the malnutrition group. In addition, the expression level of AMPK in the malnutrition group increased in both muscles. Conversely, the expression level of SIRT1 decreased in both muscles of the malnutrition group. In addition, malnutrition resulted in a decrease in the expression levels of PGC‐1α and PINK protein, and induced a decrease in the levels of two key mitochondrial enzymes (succinate dehydrogenase and citrate synthase) and COX IV protein expression in both muscles. These results indicate that malnutrition impaired the metabolic capacity in both fast and slow muscles via AMPK‐independent SIRT1 inhibition induced by increased oxidative stress.

## Graphical abstract

Malnutrition impaired mitochondrial metabolic capacity via downregulating SIRT1 induced by increased oxidative stress in both fast and slow muscles although no atrophy was observed in slow muscle. The energy‐sensing in malnutrition was characterized by AMPK‐independent SIRT1 inhibition in both fast and slow muscles.

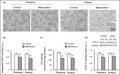

## 1 INTRODUCTION

Malnutrition, which is associated with a lack of adequate calories, protein, or other nutrients in the body, leads to the loss of body weight, muscles, and/or adipose tissues (White et al., 2012). The effects of malnutrition on skeletal muscle result in not only the loss of muscle mass but also fatigue intolerance (Lopes et al., 1982; Ruiz‐Rosado et al., 2013) owing to the reduced metabolic capacity of skeletal muscles. The effects of malnutrition on skeletal muscles differ between fast and slow muscles. Malnutrition results in a preferential loss of fast muscle mass compared to slow muscle mass (Gardiner et al., 1980; Kim, 2013).

The metabolic capacity of skeletal muscles depends on the activities of mitochondrial enzymes in the muscle fibers, which are significantly affected by the mitochondrial state (Holloszy, 1967). Low‐energy dietary intake inhibits mitochondrial metabolism owing to the decreased activities of the enzymes (Ardawi et al., 1989; Briet & Jeejeebhoy, 2001; Madapallimattam et al., 2002). Slow muscle contains many mitochondria, more fatigue resistant, and has a high metabolic capacity (Takekura & Yoshioka, 1990). Thus, fatigue intolerance associated with malnutrition may be affected by the decreased metabolic capacity of slow muscle than fast muscle. However, the differences in the metabolic capacity of fast and slow muscles in malnutrition remain largely unknown.

In chronic malnutrition, oxidative stress increases due to a decrease in albumin, which can act as an antioxidant (Fechner et al., 2001). Malnutrition with hypoalbuminemia has been reported to increase oxidative stress in the plasma and liver (Fechner et al., 2001; Manary et al., 2000; van Zutphen et al., 2016). Excessive oxidative stress damages mitochondrial DNA (Nissanka & Moraes, 2018) resulting in impaired mitochondrial function. Thus, malnutrition may increase oxidative stress in skeletal muscle owing to skeletal muscle is rich in mitochondria. In addition, some energy‐sensing factors may be involved in the reduced metabolic capacity of skeletal muscle in malnutrition. AMP‐activated protein kinase (AMPK) and silent information regulator of transcription 1 (SIRT1) act as intracellular energy‐sensing factors and regulate metabolic responses based on the energy state. The effects of malnutrition on the energy‐sensing response of AMPK and SIRT1 are also unknown.

The purposes of this study were to 1) investigate the differences in the metabolic capacity of fast and slow muscles under malnourished condition, and 2) examine the changes in oxidative stress and the energy‐sensing of AMPK/SIRT1 in malnutrition. To understand the effects of malnutrition on skeletal muscles, the activities of mitochondrial enzymes, and the expression of cytochrome c oxidase subunit 4 (COX IV) protein in the plantaris muscle (fast muscles) and soleus muscle (slow muscles) were investigated. We also analyzed the changes in the levels of AMPK and SIRT1 proteins owing to their functions as major energy‐sensing factors, peroxisome proliferator‐activated receptor γ coactivator 1‐alpha (PGC‐1α) and PTEN‐induced putative kinase protein 1 (PINK1), as the regulators of mitochondrial biogenesis or mitophagy, along with oxidative stress.

## 2 MATERIALS AND METHODS

### 2.1 Experimental animals

Twelve adult male Wistar rats (body weight 454 ± 6 g [mean ± SEM], Japan SLC) were used in this study. The rats were housed in a temperature‐controlled room at 22 ± 2°C with a light–dark cycle of 12 h and had ad libitum access to food and water. The rats were assigned randomly to the control (*n* = 6) and malnutrition groups (*n* = 6) after a week of acclimatization. The rats in the control group had ad libitum access to the standard diet (AIN‐93 M based) for 12 weeks. The malnutrition group was provided with a low‐protein diet for 12 weeks, and daily food intake was limited to 50% of the spontaneous intakes measured during the acclimatization period, in accordance with a protocol published in a previous study (Walrand et al., 2000). AIN‐93 M‐based diet, which is a low‐protein diet, was administered with a total protein content adjusted to 5% (Table 1). In the present study, both calorie restriction and a low‐protein diet induced marasmic kwashiorkor, which is a mixed‐type of marasmus (characterized by energy deficiency) and kwashiorkor (characterized by protein deficiency).

**TABLE 1:** Composition of the experimental diets.

Ingredient	Standard diet	Low protein diet
	g/kg diet	
Cornstarch	500.686	591.786
Casein	140.000	50.000
Maltodextrin	90.000	90.000
Sucrose	100.000	100.000
Soybean oil	70.000	70.000
Cellulose	50.000	50.000
Mineral mix	35.000	35.000
Vitamin mix	10.000	10.000
l‐cystine	1.800	0.700
Choline bitartrate	2.500	2.500
tert‐Butylhydroquinone	0.014	0.014
Total energy (kcal/g)	4.15	4.15

This study was approved by the Institutional Animal Care and Use Committee and performed according to the Kobe University Animal Experimentation Regulations. All experimental procedures and animal care were performed in accordance with the National Institutes of Health (NIH) Guidelines for Care and Use of Laboratory Animals (National Research Council, 1996).

### 2.2 Locomotor activity evaluation

The locomotor activity of each rat was evaluated in plastic cages (23 × 37 × 19 cm) every 2 weeks for 18 h (from 13:00 to 7:00) a day with photobeam interruption sensors (LOCOMO LS‐8; Melquest).

### 2.3 Sample preparation

After 12 weeks of the experimental period, all animals were anesthetized by injecting pentobarbital sodium (50 mg/kg body weight, *i*.*p*.). Subsequently, the plantaris and soleus muscles and the liver and epididymal adipose tissue were removed and weighed. Blood samples were collected from the inferior vena cava and centrifuged at 3,000 *g* for 10 min at 4°C. The plasma samples were collected, and the muscle samples were immediately frozen in isopentane with dry ice. The muscle and plasma samples were stored at −80°C until further histological and biochemical analyses were performed.

### 2.4 Plasma metabolite analysis

The biochemical profile of the plasma samples was analyzed using commercially available kits. The albumin and total protein concentrations in the plasma were measured by the BCG method and biuret test (A/G B‐Test Wako; Wako), respectively. The triglyceride concentrations were measured using the GPO‐DAOS method (Triglyceride E‐Test Wako; Wako). Non‐esterified fatty acid (NEFA) levels were measured using the ACS‐ACOD method (NEFA C‐Test Wako; Wako).

### 2.5 Histological analysis

The muscle samples were cut into 10‐μm sections from the middle region of the muscle belly using a cryostat (CM‐1510S, Leica Microsystems) at −25°C and then mounted on glass slides. The sections were stained with hematoxylin–eosin and succinate dehydrogenase (SDH) staining methods.

Hematoxylin–eosin staining was used to determine the cross‐sectional area (CSA) of the muscle fibers. A total of 1,200 fibers per group were analyzed from each sample (200 fibers per muscle sample). The sections were evaluated using the ImageJ software program (NIH).

SDH stain was used to determine SDH activity, as per previously described methods (Nagatomo et al., 2012). Briefly, the sections were incubated in 0.1 M phosphate buffer (pH 7.6) containing 0.9 mM NaN_3_, 0.9 mM 1‐methoxyphenazine methylsulfate, 1.5 mM nitroblue tetrazolium, and 5.6 mM EDTA–disodium salt, and 48 mM succinate disodium salt. SDH activity was also analyzed using the Image J software program, and the integrated SDH activity was calculated using a previously described method (Bekedam et al., 2003).

### 2.6 Western blot analysis

Portions (~20 mg) of the plantaris and soleus muscles were homogenized in a buffer containing 20 mM Tris–HCl (pH 7.5), 1% NP‐40, 1% sodium deoxycholate, 1 mM EDTA, 1 mM EGTA, 150 mM NaCl, 1% (v/v) protease for mammalian tissue (Sigma‐Aldrich), and 1% (v/v) phosphatase inhibitor cocktail for mammalian tissue (Sigma‐Aldrich). The homogenates were centrifuged at 15,000 *g* for 15 min at 4°C. The total protein concentration was determined using a protein determination kit (Bio‐Rad). The homogenates were solubilized in a sample loading buffer (50 mM Tris–HCl pH 6.8, 2% sodium dodecyl sulfate, 10% glycerol, 5% β‐mercaptoethanol, and 0.005% bromophenol blue) and boiled for 10 min at 80°C.

The protein samples (30 μg/lane) were separated by SDS polyacrylamide gel electrophoresis and then transferred to polyvinylidene fluoride membranes. The membranes were blocked at room temperature for 60 min with Tris‐buffered saline with Tween 20 containing 3% bovine serum albumin. After blocking, the membranes were incubated with anti‐superoxide dismutase (SOD)‐2 (1:1000 in TBST, #13141; Cell Signaling Technology), anti‐AMPKα (1:1000 in TBST, #2532; Cell Signaling Technology), anti‐phosphorylated Thr172‐AMPKα (1:1000 in TBST, #2531; Cell Signaling Technology), anti‐SIRT1 (1:1000 in TBST, #9475; Cell Signaling Technology), anti‐PGC‐1α (1:200 in TBST, sc‐13067; Santa Cruz Biotechnology), anti‐PINK1 (1:200 in TBST, sc‐517353; Santa Cruz Biotechnology), and anti‐COX IV antibodies (1:1000 in TBST, #4850; Cell Signaling Technology) overnight at 4°C. The membranes were then incubated for 60 min at room temperature with anti‐mouse or anti‐rabbit IgG conjugated to horseradish peroxidase (GE Healthcare). The proteins were detected using the chemiluminescent reagent (EzWestLumi One; ATTO). Finally, images were captured using the LAS‐1000 (Fujifilm) chemiluminescent image analyzer and quantified using the Multi‐Gauge Image Analysis Software program (Fujifilm). GAPDH (1:1000 in TBST, #97166; Cell Signaling Technology) was used as an internal control.

### 2.7 Citrate synthase activity analysis

Citrate synthase (CS) activity was analyzed as per previously described methods (Srere, 1963). Briefly, the supernatants were solubilized in a reaction buffer containing 0.1 mM DTNB and 0.3 mM acetyl‐CoA. The reaction was initiated by incubating with oxaloacetic acid (0.5 mM final concentration). The absorbance was measured at 412 nm for 5 min.

### 2.8 Oxidative stress analysis

Superoxide levels (O_2_^−^) in the plasma were analyzed using 2‐methyl‐6‐p‐methoxyphenylethynyl‐imidazopyrazinoe (MPEC) (ATTO, Tokyo, Japan) according to the manufacturer's instructions. MPEC is an imidazopyrazinone derivative that has high sensitivity and can be used to measure O_2_^−^ (Shimomura et al., 1998). Relative light units (RLU) were measured with a luminometer (Varioskan LUX; Thermo Fisher Scientific).

SOD activity was measured in the plasma using the SOD Assay Kit‐WST (Dojindo Molecular Technologies) according to the manufacturer's instructions. This kit uses WST‐1 (2‐[4‐iodophenyl]‐3‐[4‐nitrophenyl]‐5‐[2,4‐disulfophenyl]‐2H‐tetrazolium, monosodium salt) that produces a water‐soluble formazan dye upon reduction with O_2_^−^. SOD activity was determined by the inhibition of the formation of the water‐soluble formazan, which was evaluated by measuring the absorbance at 450 nm.

The levels of lipid peroxidation in muscles were analyzed by evaluating malondialdehyde (MDA) concentrations using a TBARS assay kit (Oxford Biomedical Research) according to previously described methods (Iwamura et al., 2016).

### 2.9 Statistical analyses

All values are expressed as mean ± SEM. Significant differences in body weight and locomotor activity were determined using a paired two‐way ANOVA. Significant differences between the control and malnutrition groups were analyzed using the unpaired Student's *t* test. Statistical significance was set at *p* < 0.05.

## 3 RESULTS

### 3.1 Changes in body weight and locomotor activity

**FIGURE 1:**
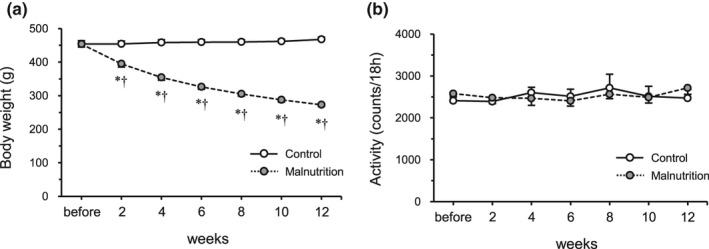
Body weight and locomotor activity. Time course of changes in body weight (a) and locomotor activity (b) during the malnourished state. The control group (*n* = 6) and malnutrition group (induced by a low‐protein diet and limited 50% diet) (*n* = 6). Values indicate mean ± SEM. * and † indicate a significant difference compared with that of the control group at the same time point, and before the same intervention, respectively (*p* < 0.05).

Body weight was lower after 2, 4, 6, 8, 10, and 12 weeks following the administration of diet inducing malnutrition in rats, and the body weights of the rats in the malnutrition group were lower than those of the rats in the control group at each point in the same week (Figure 1a). Locomotor activity was not affected by the malnourished condition (Figure 1b).

### 3.2 Muscle wet weight, muscle fiber CSA, liver weight, and epididymal adipose tissue weight

The wet weight and muscle fiber CSA of the plantaris muscle in the malnutrition group were significantly lower than those in the control group. However, the soleus muscle wet weight and muscle fiber CSA remained unchanged under malnourished condition. These results indicated that malnutrition induced the loss of the plantaris muscle mass but not of the soleus muscle. The weights of the liver and epididymal adipose tissue in the malnutrition group were significantly lower than those in the control group (Table 2).

**TABLE 2:** Muscle wet weight, muscle fiber CSA, liver weight, and epididymal adipose tissue weight.

	Control	Malnutrition
Plantaris muscle		
Muscle wet weight (mg)	402 ± 7	305 ± 10*
Muscle fiber CSA (µm^2^)	3732 ± 220	2813 ± 163*
Soleus muscle		
Muscle wet weight (mg)	150 ± 5	145 ± 6
Muscle fiber CSA (µm^2^)	3708 ± 201	3428 ± 161
Liver weight (g)	15.4 ± 0.3	8.2 ± 0.5*
Epididymal adipose weight (g)	16.7 ± 0.8	2.1 ± 0.3*

Values indicate mean ± SEM. Control group (*n* = 6) and malnutrition group (induced by a low‐protein diet and limited 50% diet) (*n* = 6). * indicates a significant difference compared with that of the control group (*p* < 0.05).

### 3.3 Levels of albumin, total protein, triglyceride, NEFA, and oxidative stress in the plasma

The albumin and total protein concentrations in the plasma were significantly lower in the malnutrition group than those in the control group. The triglyceride and NEFA concentrations in the plasma were also significantly lower in the malnutrition group than those in the control group.

The plasma O_2_^−^ levels were significantly higher in the malnutrition group than those in the control group; however, SOD activity was not significantly different between both groups (Table 3).

**TABLE 3:** Levels of albumin, total protein, triglyceride, NEFA, and oxidative stress in the plasma

	Control	Malnutrition
Albumin (g/dl)	4.4 ± 0.1	3.1 ± 0.1*
Total protein (g/dl)	6.3 ± 0.1	5.2 ± 0.1*
Triglyceride (mg/dl)	123.7 ± 27.1	44.5 ± 4.8*
NEFA (mEq/L)	1.9 ± 0.3	0.4 ± 0.1*
O_2_^−^ formation (RLU × 10^4^ counts/min)	2.8 ± 0.3	4.1 ± 0.3*
SOD activity (Unit/ml)	992 ± 88	845 ± 39

Values indicate mean ± SEM. Control group (*n* = 6) and malnutrition (induced by a low‐protein diet and limited 50% diet group) (*n* = 6). * indicates a significant difference compared with that of the control group (*p* < 0.05).

### 3.4 Change in the energy‐sensing mechanism of AMPK and SIRT1 in skeletal muscles

**FIGURE 2:**
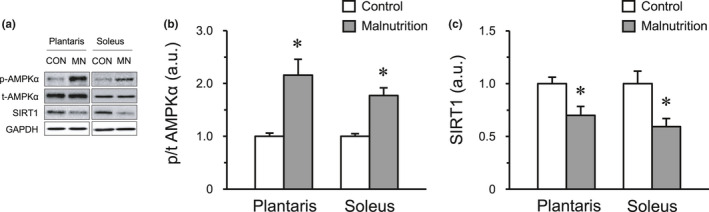
Expression of AMPKα and SIRT1 protein. Representative protein expression of phosphorylated‐AMPKα, total‐AMPKα and SIRT1 by western blotting (a). The expression levels of AMPKα and SIRT1 protein in the plantaris (b) and soleus muscle (c). The control group (CON, *n* = 6) and malnutrition group (induced by a low‐protein diet and limited 50% diet) (MN, *n* = 6). Values indicate mean ± SEM. * indicates a significant difference compared with that of the control group (*p* < 0.05).

The ratios of phosphorylated AMPKα to total AMPKα protein were significantly higher in both the plantaris and soleus muscles of the malnutrition group than those in the control group. However, the expression of SIRT1 protein in both muscles was significantly lower in the malnutrition group than that in the control group (Figure 2). These results suggest that malnutrition decreases the levels of SIRT1 independent of AMPK expression in skeletal muscle.

### 3.5 Expression of PGC‐1α and PINK1 in skeletal muscles

**FIGURE 3:**
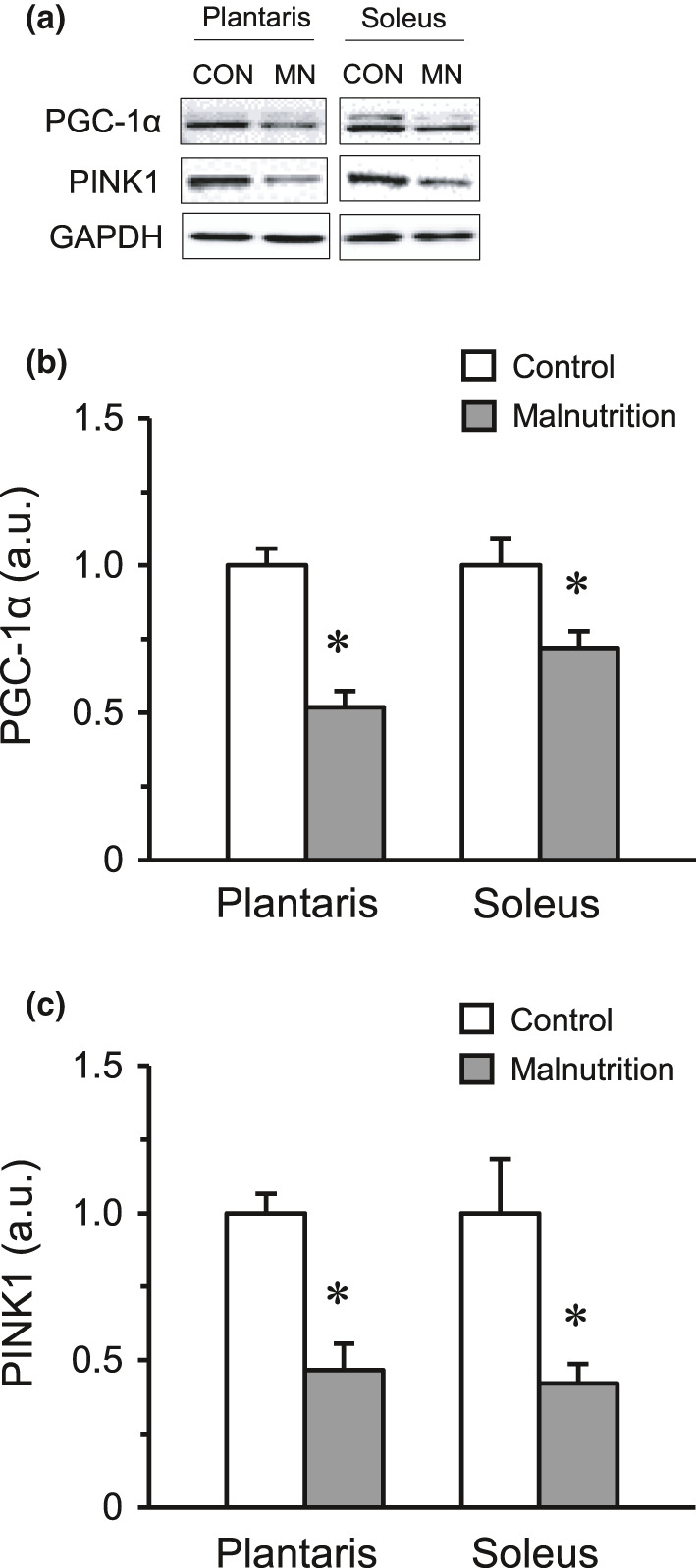
Expression of PGC‐1α and PINK1 protein. Representative protein expression of PGC‐1α and PINK1 by western blotting (a). The expression levels of PGC‐1α (b) and PINK1 (c) proteins in the plantaris and soleus muscles. The control group (CON, *n* = 6) and malnutrition group (induced by a low‐protein diet and limited 50% diet) (MN, *n* = 6). Values indicate mean ± SEM. * indicates a significant difference compared with that of the control group (*p* < 0.05).

The expression levels of PGC‐1α and PINK1 protein were significantly lower in both the plantaris and soleus muscles of the malnutrition group than those in the control group (Figure 3).

### 3.6 Mitochondrial enzyme activities and COX IV protein expression in skeletal muscles

**FIGURE 4:**
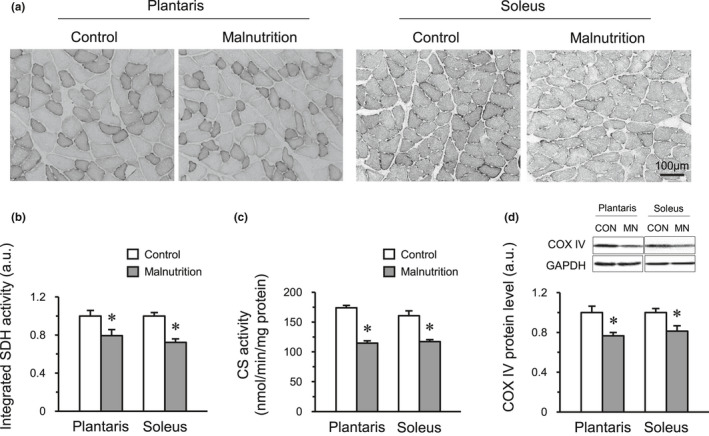
Mitochondrial enzyme activities and expression of COX IV protein. The representative region from a cross‐section of the plantaris and soleus muscle stained for SDH activity (a). The activity levels of integrated SDH (b) and CS (c) in the plantaris and soleus muscles. The expression levels of COX IV protein (d). The control group (CON, *n* = 6) and malnutrition group (induced by a low‐protein diet and limited 50% diet) (MN, *n* = 6). Values indicate mean ± SEM. * indicates a significant difference compared with that of the control group (*p* < 0.05).

The integrated SDH and CS activities were significantly lower in both the plantaris and soleus muscles of the malnutrition group than those in the control group (Figure 4b, c). The expression level of COX IV protein was significantly lower in the malnutrition group than that in the control group in both muscles (Figure 4d). These results suggest that malnutrition impairs mitochondrial metabolic capacity in both fast and slow muscles.

### 3.7 Levels of oxidative stress in skeletal muscles

**FIGURE 5:**
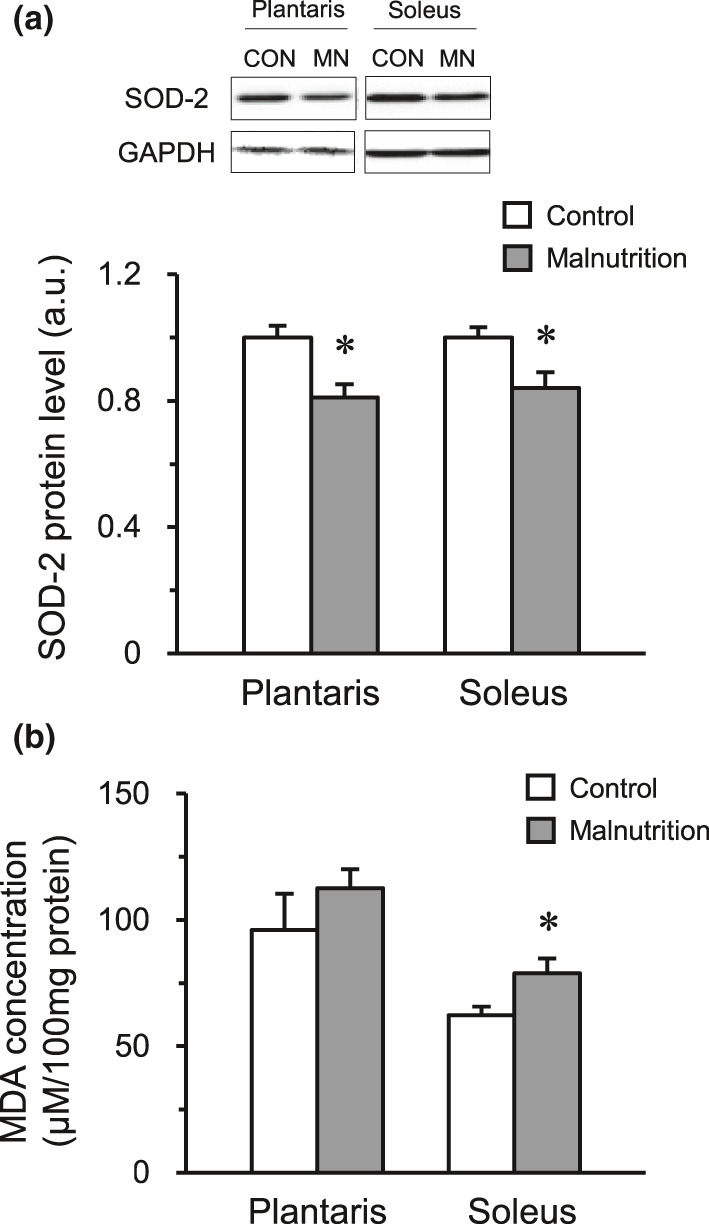
Levels of oxidative stress in skeletal muscle. The expression levels of SOD‐2 protein (a) and MDA concentration (b) in the plantaris and soleus muscle. The control group (CON, *n* = 6) and malnutrition group (induced by a low‐protein diet and limited 50% diet) (MN, *n* = 6). Values indicate mean ± SEM. * indicates a significant difference compared with that of the control group (*p* < 0.05).

The expression level of SOD‐2 protein was significantly lower in both the plantaris and soleus muscles of the malnutrition group than that in the control group. In the soleus muscle, the MDA concentrations in the soleus muscles of the malnutrition group were significantly higher than those in the control group, whereas those in the plantaris muscles were not significantly different between both groups (Figure 5).

## 4 DISCUSSION

The novel findings of the present study are listed as follows: (1) malnutrition resulted in the muscle loss of fast muscle, but no atrophy was observed in slow muscle. Regardless of this, both muscles also had metabolic disorders, (2) an increase in oxidative stress was observed in the plasma and both muscles under malnourished condition, (3) the energy‐sensing response was observed as AMPK‐independent SIRT1 inhibition in both muscles, and (4) malnutrition decreased the expression of PGC‐1α and PINK1 in both muscles. These results suggest that malnutrition impairs the metabolic capacity of fast and slow muscles via increased oxidative stress and inhibiting SIRT1.

The loss of muscle mass and decreased cross‐sectional area in the plantaris muscle were observed in the malnutrition group, but no atrophy was observed in the soleus muscle. Several previous studies reported that excessive dietary restriction or ingestion of a low‐protein diet results in loss of muscle mass in fast muscles (Kim, 2013; Pereyra‐Venegas et al., 2015; Ruiz‐Rosado et al., 2013; Toyoshima et al., 2014) and no atrophy was induced in slow muscles (Alaverdashvili et al., 2015; Salles et al., 2014; Walrand et al., 2000). In addition, Sakaida et al., (1987) reported that glycogen content in muscle fibers decreased predominantly in type IIB fibers after 2 days of starvation and almost disappeared after 4 days. Thus, malnutrition results in a major impact on fast muscles. In contrast, slow muscles are susceptible to inactivity (Ohira et al., 1994) and less susceptive to nutrition (Salles et al., 2014). In fact, the levels of locomotor activity did not change in the malnourished rats in the present study. Therefore, it was indicated that malnutrition without inactivity induces the loss of fast muscle mass but no atrophy in slow muscle.

The levels of mitochondrial enzymes, that is, CS and SDH activities, and expression of COX IV protein, decreased in both plantaris and soleus muscles of the malnutrition group. A number of studies reported that mitochondrial enzyme activities in skeletal muscle decrease by excessive dietary restriction or ingestion of a low‐protein diet (Ardawi et al., 1989; Briet & Jeejeebhoy, 2001; Madapallimattam et al., 2002; Oldfors & Sourander, 1986; Salles et al., 2014). For example, Oldfors & Sourander, (1986) reported that the ingestion of a low‐protein diet (1.5% protein) for 14 weeks decreased CS and SDH activities in both fast and slow muscles. In contrast, Faure et al., (2013) showed that a dietary restriction for 12 weeks (50% spontaneous) did not change mitochondrial enzyme activities in skeletal muscle. The metabolic capacity of skeletal muscle may be affected by a low‐protein diet than dietary restriction alone. In fact, Briet & Jeejeebhoy, (2001) reported that the ingestion of a low‐protein diet decreases mitochondrial complex activity in skeletal muscle, followed by it restored by protein refeeding but not glucose. These results indicate that malnutrition with a low‐protein diet reduces mitochondrial metabolic capacity in both fast and slow muscles.

Albumin, which accounts for approximately 60% of the protein content in the serum, is known to act as an antioxidant in blood vessels (Taverna et al., 2013). Previous studies have reported an association between increased oxidative stress and hypoalbuminemia in patients with kwashiorkor (characterized by protein deficiency) (Fechner et al., 2001; Manary et al., 2000). van Zutphen et al., (2016) reported that low protein diet‐fed rats developed hypoalbuminemia and increased hepatic malondialdehyde levels. In the present study, the level of albumin concentration decreased and the level of O_2_^−^ increased in the plasma of malnourished rats. Thus, hypoalbuminemia may be a trigger for increased oxidative stress in malnutrition. In addition, the level of SOD‐2 protein expression decreased in the plantaris and soleus muscles, and the level of the malondialdehyde concentration increased in the soleus muscle in the malnutrition group. In contrast, previous studies reported that the levels of SOD‐2 mRNA and malondialdehyde in gastrocnemius muscle did not alter after 24 hours of fasting (Qi et al., 2014) and also the level of SOD activity in the plantaris and soleus muscles did not alter after 18 days of fasting (Lammi‐Keefe et al., 1981). To the best of our knowledge, previous studies have not reported an increase in oxidative stress in skeletal muscle under an acute malnourished condition. Our study also demonstrates that chronic malnutrition with hypoalbuminemia results in increased oxidative stress in skeletal muscle.

AMPK is located upstream of SIRT1 and contributes to energy production via PGC‐1α by activating SIRT1 (Cantó et al., 2009). In the present study, malnutrition increased the level of phosphorylated AMPK expression in both the plantaris and soleus muscles. However, the expression of SIRT1 protein decreased despite an increase in AMPK expression in both muscles of the malnutrition group. We focused on the effects of oxidative stress on the discrepancy between the expression levels of AMPK and SIRT1 under malnourished condition. Several previous studies showed that oxidative stress activates AMPK (Auciello et al., 2014; Hinchy et al., 2018; Zmijewski et al., 2010) but inhibits SIRT1 (Chen et al., 2013; Liang et al., 2020). Zmijewski et al., (2010) reported that exposure of HEK 293 cells to H_2_O_2_ resulted in the activation of AMPK that was dose‐dependent. Also, Liang et al., (2020) showed that the expression of SIRT1 protein was downregulated in porcine intestinal epithelial cells upon treatment with H_2_O_2_. Thus, the response to ROS differs between AMPK and SIRT1. These results suggest that the energy‐sensing response of skeletal muscle in malnutrition was characterized by AMPK‐independent SIRT1 inhibition induced by increased oxidative stress.

PGC‐1α is located downstream of SIRT1 and is activated by the deacetylation of SIRT1 to upregulate mitochondrial biogenesis (Lin et al., 2005; Tang, 2016). In fact, ingestion of resveratrol (representative of SIRT1 activator) has been shown to increase PGC‐1α protein expression and mitochondrial DNA content in the gastrocnemius muscle (Lagouge et al., 2006). In the present study, the levels of SIRT1 and PGC‐1α protein expression decreased in the plantaris and soleus muscles of malnourished rats. Thus, malnutrition‐induced downregulation of the SIRT1/PGC‐1α pathway may impair mitochondrial biogenesis in skeletal muscle. In addition, PINK1 is induced by increased oxidative stress and initiates mitophagy (Kitagishi et al., 2017). In fact, H_2_O_2_ treatment on the porcine intestinal epithelial cells has been shown to increase PINK1 protein (Liang et al., 2020). However, the level of PINK1 protein expression decreased despite increased oxidative stress due to malnutrition in the present study. These results suggest that PINK1 may be regulated by factors other than oxidative stress. Several previous studies have shown an association between mitophagy and SIRT1 (Jang et al., 2012; Liang et al., 2020; Qiao et al., 2018; Yao et al., 2018). Yao et al., (2018) showed that knockdown of SIRT1 resulted in a decrease in PINK1 protein in the U87MG and T98G cells. These results suggest that the decreased SIRT1 in malnutrition might be induced by the downregulation of PINK1, an upstream factor of mitophagy. Moreover, previous studies showed that PGC1α and PINK1 affect mitochondrial metabolic capacity in the skeletal muscle (Gautier et al., 2008; Zechner et al., 2010). Zechner et al., (2010) reported that the levels of SDH activities and COX IV mRNA decreased in the PGC‐1α knockout mice. Also, Gautier et al., (2008) reported that the level of some complex respiratory activities decreased in the PINK1 knockout mice owing to increased oxidative stress sensitivity of mitochondria. Thus, the decrease in PGC‐1α and PINK1 is also a factor that reduces the mitochondrial metabolic capacity. Together our results indicate that malnutrition‐induced downregulation of SIRT1 impairs mitochondrial homeostasis and metabolic capacity in skeletal muscle through decreased PGC‐1α and PINK1. Furthermore, these results were suggested that changes in oxidative stress and SIRT1 were reduced metabolic capacity even under malnourished condition without the atrophy of slow muscles. Meanwhile, these results suggest that changes in oxidative stress and SIRT1 might decrease the metabolic capacity of slow muscle under malnourished condition without the atrophy.

A limitation of this study is that we determined only PGC‐1α and PINK1 protein levels to assess mitochondrial biogenesis and mitophagy in the present study. Future studies should be performed to determine the downstream factors of PGC‐1α and PINK1 or other pathways of mitochondrial homeostasis in malnutrition.

## 5 CONCLUSIONS

This study demonstrated that malnutrition impaired the metabolic capacity of both fast and slow muscles. In addition, the energy‐sensing response of both muscles in malnutrition was characterized by AMPK‐independent SIRT1 inhibition induced by increased oxidative stress. Abnormalities in the energy‐sensing response in malnutrition could impair mitochondrial homeostasis. These findings may play a key role in understanding the energy metabolism of skeletal muscle under malnourished condition.

## CONFLICT OF INTEREST

None declared.

## AUTHOR CONTRIBUTIONS

T. H. and H. F. conceived the study, participated in study design, managed data collection, conducted the statistical analysis, and drafted and revised the manuscript. R. N., M. T., and B. N. participated in data collection and data analysis. N. M. and H. K. reviewed and revised the manuscript. All authors read and approved the final manuscript.
